# Mesoporous Silica Nanoparticles Impair Physiology and Reproductive Fitness of *Tuta absoluta* Through Plant-Mediated Oxidative Stress and Enzymatic Disruption

**DOI:** 10.3390/insects16090877

**Published:** 2025-08-23

**Authors:** Inzamam Ul Haq, Huiping Liu, Muhammad Adeel Ghafar, Saba Zafar, Mishal Subhan, Asim Abbasi, Moazam Hyder, Abdul Basit, Nazih Y. Rebouh, Youming Hou

**Affiliations:** 1 State Key Laboratory of Agricultural and Forestry Biosecurity, Key Laboratory of Biopesticides and Chemical Biology, MOE, College of Plant Protection, Fujian Agriculture and Forestry University, Fuzhou 350002, China; 2 Department of Biochemistry and Biotechnology, The Women University Multan, Multan 66000, Pakistan; 3 Department of Microbiology and Molecular Genetics, The Women University Multan, Multan 66000, Pakistan; 4 Department of Entomology, University of Agriculture, Faisalabad 38040, Pakistan; asimuaf95@gmail.com; 5 Department of Environmental Management, Institute of Environmental Engineering, RUDN University, 6 Miklukho-Maklaya St., Moscow 117198, Russia

**Keywords:** *Tuta absoluta*, mesoporous silica nanoparticles, tomato plant antioxidants, insect digestive enzymes, nanobiopesticides, integrated pest management (IPM)

## Abstract

The tomato leafminer (*Tuta absoluta*) is a globally invasive pest that causes severe yield losses in tomato crops. Nanotechnology-based strategies offer promising alternatives to conventional insecticides. This study examines the physiological, biochemical, and demographic responses of *T. absoluta* following exposure to mesoporous silica nanoparticles (MSNs) applied to tomato leaves at concentrations of 0, 3, 30, and 300 mg L^−1^. Comprehensive assessments were conducted, including digestive and detoxifying enzyme activities in the insect, neurotoxicity indicators, life table parameters, and antioxidant responses in the host plant. At 30 mg L^−1^, MSNs significantly impaired larval development, fecundity, and survival of *T. absoluta* without inducing phytotoxicity. Tomato plants treated at this concentration exhibited enhanced antioxidant enzyme activity (SOD, CAT, POD) and a reduced malondialdehyde (MDA) content, indicating an active oxidative defense. These plant responses were significantly correlated with changes in insect fitness traits, suggesting a plant-mediated effect on pest physiology. Digestive enzyme disruption, decreased acetylcholinesterase activity, and extended developmental periods contributed to suppressed population growth, as evidenced by reductions in the intrinsic rate of increase (r), net reproductive rate (R_0_), and fecundity. At 300 mg L^−1^, however, severe phytotoxicity and enzymatic collapse were observed in both plant and insect systems. These findings highlight moderate concentration of MSNs (30 mg L^−1^) as a promising dose for sustainable and host-safe pest management, offering multi-targeted suppression of *T. absoluta* through combined plant and insect biochemical pathways.

## Simple Summary

The tomato is a vital vegetable crop grown worldwide, but its productivity is threatened by *Tuta absoluta*, a small insect that damages tomato leaves and fruits, resulting in significant yield losses. While farmers often rely on chemical pesticides to control this pest, excessive use can harm the environment and cause pesticide resistance. In this study, we tested tiny, plant-safe mesoporous silica nanoparticles (MSNs) on tomato leaves and tracked how the tomato leafminer (*T. absoluta*) responded. At 30 mg L^−1^, plants showed healthier physiology and the pest’s growth and reproduction dropped; at 300 mg L^−1^, both plant and insect were stressed. MSNs worked indirectly through the plant (antioxidant defenses) and directly on the insect (digestive and neural enzymes), pointing to a practical, lower-dose option that could reduce reliance on conventional pesticides.

## 1. Introduction

The tomato leafminer, *Tuta absoluta* (Meyrick) (Lepidoptera: Gelechiidae), is a highly destructive pest of the tomato (*Solanum lycopersicum* L.; family Solanaceae, order Solanales) and poses a significant threat to tomato production worldwide [1,2]. Originating in South America, *T. absoluta* has rapidly expanded its range across Europe, Africa, and Asia, causing substantial economic losses in both open-field and greenhouse systems [3,4]. *T. absoluta* is a holometabolous moth whose life cycle comprises egg, four leaf-mining larval instars, pupa, and adult [3]. Eggs are laid mainly on the abaxial leaf surface; larvae mine mesophyll tissue and form characteristic galleries, while pupation occurs within mines, on foliage, or in the soil [5]. Adults are predominantly crepuscular/nocturnal and highly fecund. The developmental rate is strongly temperature-dependent, with a short generation time that enables multiple, often overlapping generations per season, facilitating rapid population build-up in both protected and open-field tomatoes [6,7]. Its high reproductive potential, cryptic larval behavior, and ability to develop resistance to multiple classes of insecticides have rendered conventional chemical control strategies increasingly ineffective [7,8,9]. As a result, there is a growing interest in developing alternative, environmentally safe pest control approaches that minimize ecological risks while ensuring sustainable agricultural productivity [10,11,12].

In recent years, nanotechnology has emerged as a promising tool for crop protection and pest management [13,14,15]. Among various engineered nanomaterials, mesoporous silica nanoparticles (MSNs) have attracted attention due to their unique physicochemical properties, including high surface area, tunable pore size, thermal stability, and biocompatibility [16,17]. These characteristics facilitate the incorporation and sustained release of bioactive agents, enhancing interactions with biological membranes and making MSNs potential candidates for use as delivery systems or standalone pest control agents [18,19,20]. While several studies have explored the pesticidal efficacy of metal-based nanoparticles such as ZnO and TiO_2_ [21,22,23], the biological impact of MSNs on insect physiology and population dynamics remains poorly understood [24].

Although the physiological impacts of MSNs on insects require further clarification, several studies indicate their utility in pest management. For example, dual-stimulus-responsive MSNs have been engineered to control *Spodoptera litura* (Lepidoptera), a key tomato pest, by coupling temperature and α-amylase triggers for on-demand release [25]. Moreover, pH-responsive MSNs have been used to deliver a trypsin inhibitor to *Helicoverpa armigera*, exploiting the alkaline lepidopteran midgut to enhance efficacy [26]. In the tomato pathosystem itself, mesoporous nanosilica supplementation altered plant defenses against the tomato pinworm *Phthorimaea absoluta* (syn. *T. absoluta*), shifting oviposition patterns and increasing caterpillar mortality in tritrophic assays [27]. Beyond tomato pests, dendritic MSNs have served as nanocarriers to deliver siRNA (and co-load an insecticide) to the rice planthopper *Sogatella furcifera*, suppressing detoxification pathways and synergizing chemical control—underscoring the broader potential of MSN platforms [28].

Insect responses to nanoparticles are often mediated through complex biochemical pathways [29]. Exposure to nanoparticles can induce oxidative stress by generating reactive oxygen species (ROS), which disrupt cellular homeostasis, damage biomolecules, and activate antioxidant defense systems [30,31,32]. Furthermore, nanoparticles may interfere with enzymatic functions essential for digestion, detoxification, and neurophysiological regulation, leading to altered development, reproduction, and survival [33,34,35]. Despite these insights, few studies have attempted to comprehensively evaluate the sublethal and chronic effects of MSNs across multiple biological levels in pest insects [36,37].

The present study was designed to bridge this knowledge gap by evaluating the biochemical, physiological, and demographic effects of mesoporous silica nanoparticles on *T. absoluta*. We synthesized and characterized MSNs, applied them at different concentrations to tomato plants, and assessed the impact on *T. absoluta* through a combination of enzyme assays, physiological measurements, and life table analyses. Specifically, we investigated changes in oxidative stress markers (MDA), antioxidant enzymes (SOD, CAT, POD), digestive enzymes (amylase, lipase), and detoxification-related enzymes (P450, AChE). These biochemical responses were then linked to alterations in fecundity, development time, survival, and reproductive fitness using age–stage, two-sex life table analysis. Additionally, correlation analysis was conducted to elucidate the relationships between biochemical stress responses and key life-history parameters. By integrating mechanistic and demographic endpoints, this study provides a holistic assessment of the biological consequences of MSN exposure in *T. absoluta* and offers new insights into the potential of MSNs as eco-friendly agents for pest management. The findings may contribute to the development of nanomaterial-based pest control strategies aligned with the goals of integrated pest management (IPM) and sustainable agriculture.

## 2. Materials and Methods

### 2.1. Synthesis and Characterization of Mesoporous Silica Nanoparticles (MSNs)

**Figure 1:**
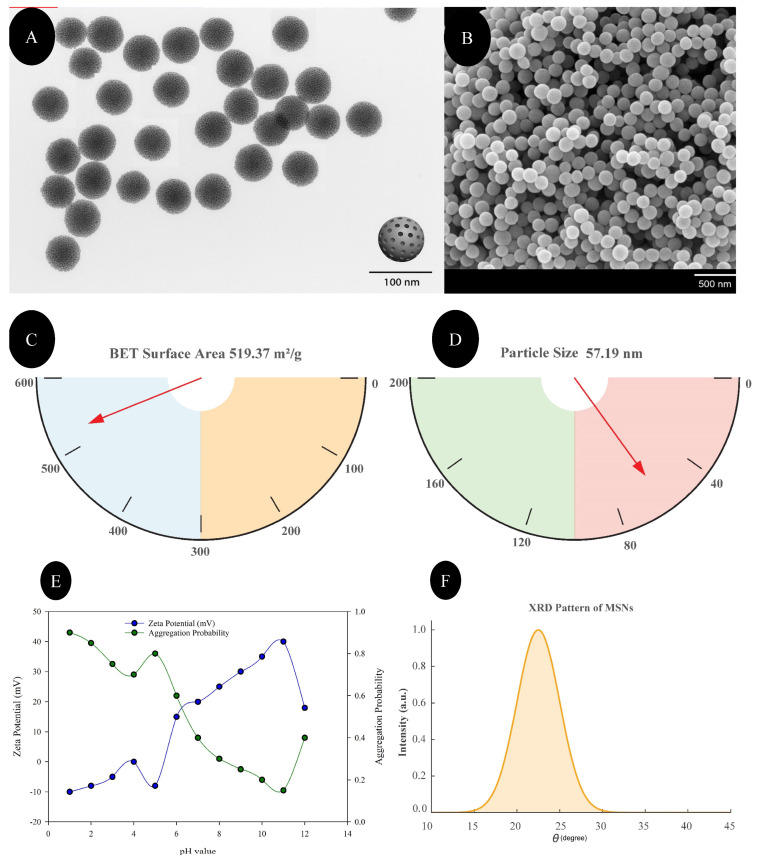
Physicochemical characterization of mesoporous silica nanoparticles (MSNs). (**A**) Transmission electron microscopy (TEM) micrograph. (**B**) Scanning electron microscopy (SEM) micrograph. (**C**) Brunauer–Emmett–Teller (BET) specific surface area (m^2^ g^−1^). (**D**) Dynamic light scattering (DLS) hydrodynamic diameter (nm). (**E**) Zeta potential (mV) and aggregation probability as a function of pH. (**F**) X-ray diffraction (XRD) pattern (arbitrary units) versus 2θ (degrees).

Mesoporous silica nanoparticles were synthesized using a sol–gel method [38]. Tetraethyl orthosilicate (TEOS) served as the silica precursor, and cetyltrimethylammonium bromide (CTAB) was used as the structure-directing agent. The reaction mixture was stirred under alkaline conditions at room temperature for several hours, followed by aging, washing, and calcination to remove the surfactant. The synthesized MSNs were characterized using transmission electron microscopy (TEM) to evaluate morphology and mesoporous structure; surface morphology was additionally inspected by scanning electron microscopy (SEM; field-emission, 5–10 kV). Particle size distribution, polydispersity index (PDI), and zeta potential were measured using dynamic light scattering (DLS) on a Zetasizer Nano ZS (Malvern Instruments, UK). Particle diameters were quantified from calibrated TEM micrographs (Figure 1A) using ImageJ software (version 1.54f; National Institutes of Health, Bethesda, MD, USA). Clearly delineated, non-overlapping particles were segmented and measured as equivalent circular diameter (ECD). For the representative field shown in Figure 1A, n = 28 particles were calculated and summarized as number-weighted statistics; these dry-state TEM sizes were compared with the hydrodynamic diameters obtained from DLS. The average hydrodynamic size and colloidal stability were recorded based on three replicate measurements.

### 2.2. Tomato Cultivation and MSN Application

Tomato plants (*S. lycopersicum* L., Solanaceae) were grown under controlled greenhouse conditions with uniform light, humidity, and temperature. At the four-leaf stage, plants were randomly assigned to four treatment groups: control (0 mg L^−1^), low (3 mg L^−1^), medium (30 mg L^−1^), and high (300 mg L^−1^) concentrations of MSNs. Concentrations (3, 30, 300 mg L^−1^) were selected a priori to span low, moderate, and high exposure levels based on preliminary range-finding and published nanoparticle studies, with 30 mg L^−1^ targeted as a sublethal, plant-tolerant dose for demographic assays. Nanoparticle suspensions were freshly prepared in deionized water and applied via foliar spray using a hand sprayer until runoff occurred. Plants received four foliar applications, administered once per week for four consecutive weeks. At the first application, plants were at the four true-leaf stage (BBCH 14) [39]. Each treatment comprised 3 biological replicates of 12 plants (36 plants per treatment). Sprays were applied once weekly for four weeks to incipient runoff with ~35 mL plant^−1^ per application, rotating plants to coat both adaxial and abaxial leaf surfaces. For life-table assays, neonates were reared individually (one larva per 90 mm dish) on a 6.2 cm leaf disc; 20 larvae per replicate (N = 60 per treatment; total N = 240), with observations every 24 h from hatch to adult emergence. Leaves for insect bioassays were excised 48 h after the application.

### 2.3. Insect Rearing and Feeding Bioassay

A laboratory colony of *T. absoluta* was maintained on untreated tomato plants at 25 ± 2 °C, 65 ± 5% RH, and a 16:8 h light: dark photoperiod. For bioassays, newly hatched larvae were transferred individually into Petri dishes (90 mm diameter) lined with moist filter paper. (See Section 2.2).

### 2.4. Biochemical Assays

All extractions were performed on ice in ice-cold phosphate buffer (50 mM, pH 7.0). Homogenates were centrifuged at 12,000× *g* for 15 min at 4 °C, and soluble protein was quantified by the Bradford method. Unless stated otherwise, activities are expressed as U mg^−1^ protein. All assays used three biological replicates per treatment.

#### 2.4.1. Plant (Leaf) Assays

Leaf tissue from MSN-treated tomato plants was sampled at the designated time points (30 and 45 days after transplanting; see Section 2.2), homogenized as above, and the supernatant used for biochemical determinations. Lipid peroxidation was quantified as MDA using the TBARS assay [40]. Antioxidant enzyme activities of superoxide dismutase (SOD), catalase (CAT), and peroxidase (POD) were measured using standard spectrophotometric protocols [41,42].

#### 2.4.2. Insect (Larval) Assays

Freshly collected *T. absoluta* larvae from each treatment were homogenized as above and the supernatant used for enzyme determinations. Digestive enzymes were assayed for protease, α-amylase (starch hydrolysis) [43], and lipase (*p*-nitrophenyl palmitate hydrolysis) [44]. Detoxification/neural enzymes—cytochrome P450, glutathione S-transferase (GST), carboxylesterase (CarE), and acetylcholinesterase (AChE)—were quantified using commercial kits (Solarbio Life Sciences, Beijing, China) according to the manufacturer’s instructions. Results were expressed as U mg^−1^ protein.

### 2.5. Life Table and Reproductive Performance

Age–stage, two-sex life table analysis was conducted using the TWOSEX-MSChart program to estimate comprehensive population parameters, including the intrinsic rate of increase (*r*), finite rate of increase (*λ*), net reproductive rate (*R*_0_), gross reproductive rate (*GRR*), mean generation time (*T*), adult pre-oviposition period (*APOP*), total pre-oviposition period (*TPOP*), and fecundity per female (*F*). Age-specific survival rates (*l_x_*), age–stage-specific survival rates (*S_xj_*), age–stage life expectancies (*E_xj_*), age–stage reproductive values (*V_xj_*), and age-specific fecundity (*f_x_*) were calculated for each treatment group. Data were collected from newly hatched larvae and tracked through all developmental stages. Female emergence and oviposition were monitored daily to construct accurate age–stage-specific schedules. Bootstrap resampling (n = 100,000) was applied to calculate the means and standard errors of all life table parameters. Preadult survivorship (*Sa*), the number of reproductive females (*RepF*), and oviposition days were also recorded. The following standard formulas were used to calculate age–stage, two-sex life table parameters [45,46]:

$$\mathrm{Age-specific}\;\mathrm{survival}\;\mathrm{rate}:\;(l_{x})=\sum_{j=1}^{m}s_{xj}$$

where, *S_xj_* is the age–stage-specific survival rate of individuals at age *x* and stage *j*.

$$\mathrm{Age-specific}\;\mathrm{fecundity}:\;\left(m_{x}\right)=\frac{\sum_{j=1}^{m}f_{xj}}{\sum_{j=1}^{m}s_{xj}}$$

where *f_xj_* is the age–stage-specific fecundity.

$$\mathrm{The}\;\mathrm{intrinsic}\;\mathrm{rate}\;\mathrm{of}\;\mathrm{increase}:\;(r)=\sum_{x=0}^{\infty }e^{-r\left(x+1\right)}l_{x}m_{x}=1$$

$$\mathrm{The}\;\mathrm{finite}\;\mathrm{rate}\;\mathrm{of}\;\mathrm{increase}:\;(\lambda )=e^{r}$$

$$\mathrm{The}\;\mathrm{net}\;\mathrm{reproduction}\;\mathrm{rate}:\;(R_{0})=\sum_{x=0}^{\infty }l_{x}m_{x}$$

$$\mathrm{The}\;\mathrm{mean}\;\mathrm{generation}\;\mathrm{time}:\;(T)=\frac{lnR_{0}}{r}$$

$$\mathrm{The}\;\mathrm{life}\;\mathrm{expectancy}:\;(e_{xj})=\sum_{i=x}^{\infty }.\sum_{y=j}^{\beta }s_{iy}$$

$$\mathrm{The}\;\mathrm{reproductive}\;\mathrm{value}:\;(v_{xj})=\frac{e^{r\left(x+1\right)}l_{x+1}\;m_{x+1}}{l_{xj}}$$

All calculations were conducted using the TWOSEX-MSChart program [47].

### 2.6. Statistical and Correlation Analysis

Data were screened for ANOVA assumptions by inspecting Q–Q plots and residuals-versus-fitted diagnostics; normality was tested with the Shapiro–Wilk test and homogeneity of variances with Levene’s test (median-based). When necessary, variables were transformed to better meet the assumptions (ln(x + 1) for enzyme/physiological measurements; arcsine square root for percentage/proportion data). If assumptions were not met, comparisons were conducted using the Kruskal–Wallis test followed by Dunn’s post hoc comparisons with the Holm adjustment; otherwise, a one-way ANOVA with Tukey’s HSD was applied. Statistical significance was set at α = 0.05 (two-tailed). For associations, Pearson correlation coefficients were computed using either raw or transformed variables as above; correlation matrices were visualized as seaborn heatmaps in Python. Correlation analyses were exploratory and reflect association, not causality. Analyses were performed in GraphPad Prism 8.0, SPSS 22.0, and Python. Correlation heatmaps were generated in Python v3.11 using seaborn v0.13; life-table bootstraps (n = 100,000) were computed in TWOSEX-MSChart (2023 release).

## 3. Results

### 3.1. Characterization of Mesoporous Silica Nanoparticles (MSNs)

Transmission electron microscopy (TEM) analysis (Figure 1A) confirmed that the synthesized mesoporous silica nanoparticles (MSNs) exhibited uniform spherical morphology with a clearly defined mesoporous internal structure. The particles were well-dispersed without visible agglomeration, indicating effective stabilization. Quantitative analysis of the TEM micrograph yielded a number-weighted diameter of 59.6 ± 6.9 nm (mean ± SD; median 59.1 nm, IQR 56.8–61.4 nm; n = 28), consistent with the mesoporous, near-spherical morphology. Scanning electron microscopy (SEM) (Figure 1B) further supported the spherical and monodisperse characteristics of the MSNs, demonstrating consistent particle size and shape. The Brunauer–Emmett–Teller (BET) analysis (Figure 1C) revealed a specific surface area of 519.37 m^2^/g, suggesting a well-developed porous network. The high surface area is favorable for enhanced biological interactions and has the potential for increased efficacy in contact-based applications.

Dynamic light scattering (DLS) analysis (Figure 1D) indicated an average particle size of 57.19 nm, validating the nanoscale dimensions of the MSNs. As expected, the DLS hydrodynamic diameter was similar (57.19 nm), reflecting particle solvation and the electrical double layer (Figure 1D). The zeta-potential shifted from ~−10 mV in acidic media to ~+40 mV at pH 10; the positive values at alkaline pH likely arise from residual cationic surface groups originating from the CTAB template, and higher zeta coincided with lower aggregation probability, indicating maximum colloidal stability near pH 9–10 (Figure 1E). The X-ray diffraction (XRD) pattern (Figure 1F) exhibited a broad peak centered around 2θ ≈ 22°, indicating the amorphous nature of the silica matrix. The absence of sharp diffraction peaks confirmed the lack of a long-range crystalline order, a characteristic of mesoporous silica synthesized via soft-templating approaches. The amorphous structure supports the chemical stability and biocompatibility of MSNs for biological applications.

### 3.2. Physiological Effects of MSNs on Tomato Plants

The application of mesoporous silica nanoparticles (MSNs) had a significant effect on chlorophyll content in tomato leaves at both 30 and 45 days after transplanting (DAT) (Figure A). At 30 DAT, chlorophyll content increased from 42.88 ± 1.67 SPAD units in the control to 46.73 ± 1.41 at 3 mg L^−1^ and peaked at 52.54 ± 2.42 at 30 mg L^−1^. However, a decline was observed at 300 mg L^−1^, where the chlorophyll content dropped to 46.74 ± 1.84. A similar pattern was noted at 45 DAT, with values increasing from 44.29 ± 2.09 (control) to 50.03 ± 2.20 (30 mg L^−1^) before decreasing to 46.76 ± 1.98 at the highest concentration. Across all treatments, chlorophyll values were slightly higher at 45 DAT compared to 30 DAT.

**Figure 2:**
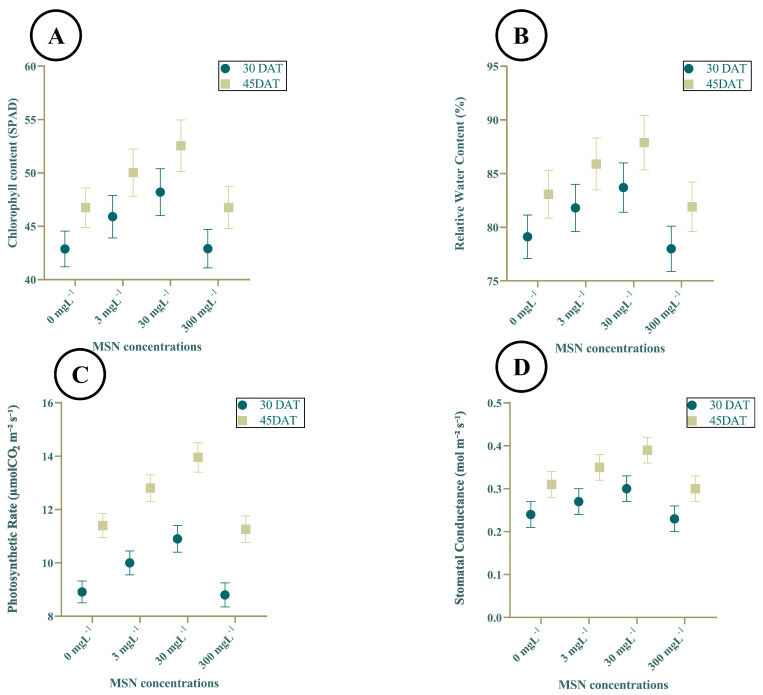
Physiological traits of tomato at 30 and 45 days after transplanting (DAT) under MSN treatments (0, 3, 30, 300 mg L^−1^). (**A**) Chlorophyll content (SPAD). (**B**) Relative water content (%). (**C**) Net photosynthetic rate (Pn; μmol CO_2_ m^−2^ s^−1^). (**D**) Stomatal conductance (gs; mol H_2_O m^−2^ s^−1^). Values are mean ± SE (n = 12 plants per replicate; three replicates per treatment).

Relative water content (RWC) also exhibited a dose-dependent response (Figure 2B). At 30 DAT, RWC increased from 79.36 ± 1.36% in the control to 85.03 ± 1.21% at 30 mg L^−1^, followed by a decline to 82.65 ± 1.04% at 300 mg L^−1^. At 45 DAT, a comparable trend was observed: RWC rose from 80.42 ± 1.45% (control) to a maximum of 88.35 ± 1.53% at 30 mg L^−1^ and declined to 83.06 ± 1.28% at 300 mg L^−1^. Across time points, the 30 mg L^−1^ treatment consistently exhibited the highest RWC values, indicating a consistent physiological response to MSNs at this concentration.

The photosynthetic rate (Pn) showed marked differences among treatments and time points (Figure 2C). At 30 DAT, the photosynthetic rate increased from 8.38 ± 0.63 μmol CO_2_ m^−2^ s^−1^ in the control to 10.57 ± 0.61 μmol CO_2_ m^−2^ s^−1^ at 3 mg L^−1^, reaching the highest value of 13.72 ± 0.65 μmol CO_2_ m^−2^ s^−1^ at 30 mg L^−1^. A subsequent decrease was observed at 300 mg L^−1^ (9.73 ± 0.67 μmol CO_2_ m^−2^ s^−1^). At 45 DAT, similar trends were observed, with values ranging from 9.02 ± 0.74 μmol CO_2_ m^−2^ s^−1^ (control) to 14.49 ± 0.86 μmol CO_2_ m^−2^ s^−1^ (30 mg L^−1^), followed by a decrease to 10.06 ± 0.77 μmol CO_2_ m^−2^ s^−1^ at 300 mg L^−1^.

Stomatal conductance (gs) also responded to MSN exposure in a concentration-dependent manner (Figure 2D). At 30 DAT, gs increased from 0.27 ± 0.01 mol m^−2^ s^−1^ in the control to 0.33 ± 0.01 mol m^−2^ s^−1^ at 3 mg L^−1^ and peaked at 0.36 ± 0.02 mol m^−2^ s^−1^ at 30 mg L^−1^, before decreasing to 0.30 ± 0.01 mol m^−2^ s^−1^ at 300 mg L^−1^. At 45 DAT, the same trend was evident, with gs values ranging from 0.30 ± 0.01 mol m^−2^ s^−1^ (control) to 0.42 ± 0.02 mol m^−2^ s^−1^ (30 mg L^−1^), followed by a decline to 0.32 ± 0.01 mol m^−2^ s^−1^ at 300 mg L^−1^. Across both time points, the 30 mg L^−1^ treatment consistently showed the highest photosynthetic and stomatal performance.

### 3.3. Antioxidant Enzyme Activities

**Figure 3:**
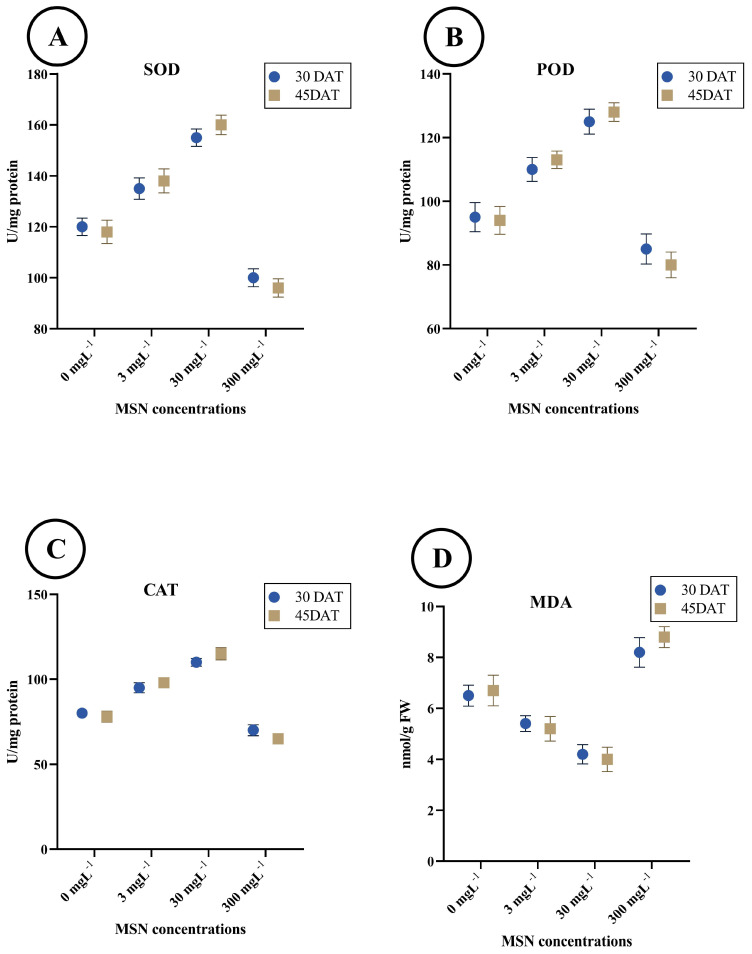
Antioxidant enzyme activities and lipid peroxidation in tomato leaves at 30 and 45 days after transplanting (DAT) under mesoporous silica nanoparticle (MSN) treatments (0, 3, 30, 300 mg L^−1^). (**A**) Superoxide dismutase (SOD; U mg^−1^ protein). (**B**) Peroxidase (POD; U mg^−1^ protein). (**C**) Catalase (CAT; U mg^−1^ protein). (**D**) Malondialdehyde (MDA; nmol mg^−1^ protein). Bars show mean ± SE (n = 3 biological replicates per treatment).

The activity of SOD increased progressively with MSN concentrations up to 30 mg L^−1^ and subsequently declined at the highest concentration (Figure 3A). At 30 DAT, SOD activity was 125.3 ± 2.8 U/mg protein in the control group, increasing to 138.6 ± 3.2 at 3 mg L^−1^ and peaking at 165.7 ± 3.6 at 30 mg L^−1^. A decrease was observed at 300 mg L^−1^, with activity reducing to 105.6 ± 2.9. A similar pattern was observed at 45 DAT, with SOD activity ranging from 122.4 ± 2.4 (control) to 160.3 ± 3.1 (30 mg L^−1^) and then decreasing to 108.2 ± 3.0 at 300 mg L^−1^.

POD activity also demonstrated a dose-dependent increase up to 30 mg L^−1^, followed by a decline at 300 mg L^−1^ (Figure 3B). At 30 DAT, values rose from 103.2 ± 2.6 U/mg protein (control) to 125.4 ± 2.9 U/mg protein (30 mg L^−1^), then decreased to 84.5 ± 2.2 U/mg protein at 300 mg L^−1^. At 45 DAT, POD activity was slightly higher across treatments, with the highest value recorded at 130.4 ± 3.1 U/mg protein for the 30 mg L^−1^ treatment and the lowest at 87.3 ± 2.6 U/mg protein for the 300 mg L^−1^ treatment.

CAT activity showed similar trends to SOD and POD (Figure 3C). At 30 DAT, activity was 88.4 ± 2.0 U/mg protein in the control, increasing to 102.3 ± 2.4 (3 mg L^−1^), and reaching 116.7 ± 2.8 at 30 mg L^−1^. The 300 mg L^−1^ treatment reduced CAT activity to 69.1 ± 1.9. At 45 DAT, the maximum CAT activity (120.2 ± 3.1 U/mg protein) was observed at 30 mg L^−1^, with a subsequent reduction to 72.4 ± 2.0 U/mg protein at the highest dose.

MDA content decreased with increasing MSN concentrations up to 30 mg L^−1^, followed by a sharp increase at 300 mg L^−1^ (Figure 3D). At 30 DAT, MDA levels declined from 6.73 ± 0.21 nmol/g FW in the control to 5.28 ± 0.18 at 30 mg L^−1^, then increased to 8.64 ± 0.24 at 300 mg L^−1^. A similar pattern was observed at 45 DAT, where MDA decreased from 6.81 ± 0.19 (control) to 5.04 ± 0.17 at 30 mg L^−1^ and rose to 8.01 ± 0.23 at the highest concentration.

### 3.4. Age–Stage-Specific Survival Rate (Sₓⱼ)

**Figure 4:**
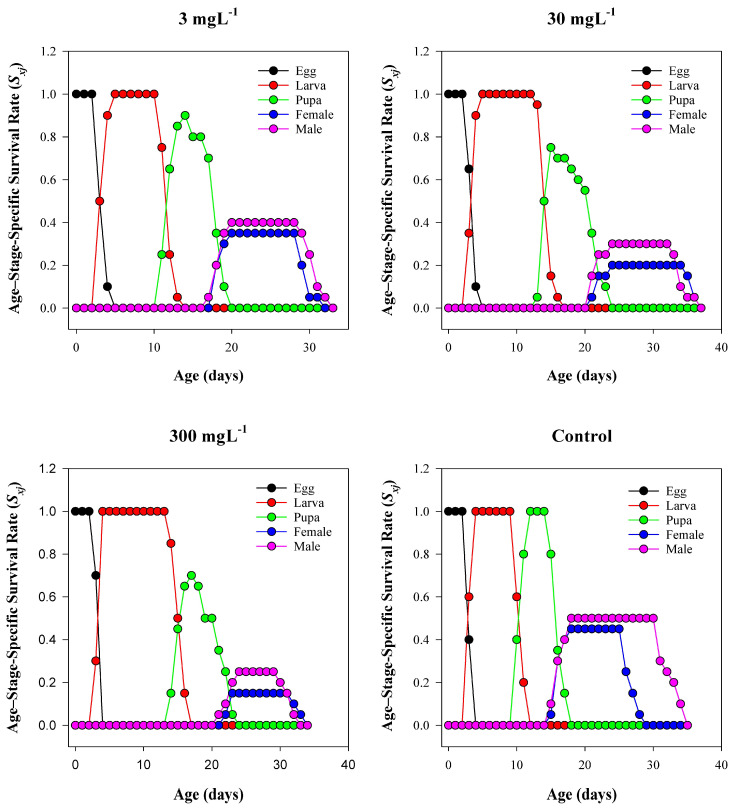
Age–stage-specific survival rate (*Sₓⱼ*) of *T. absoluta* under mesoporous silica nanoparticle (MSN) treatments (0, 3, 30, 300 mg L^−1^). Axes: x = age (days); y = *Sₓⱼ* (proportion). Stages in legend: Egg, Larva, Pupa, Female adult, Male adult.

The age–stage survival trajectories (Figure 4) showed dose-dependent compression of adult plateaus and earlier declines at higher MSN concentrations. In the control, the larval stage occupied ages of ~4–14 days with a high, flat plateau; pupae appeared around day 15, and adult cohorts (females and males) established broad plateaus from ~20 to 34 days, with females persisting slightly longer than males. At 3 mg L^−1^, the larval plateau shortened modestly, and the adult plateau began ~1–2 d later and ended ~3–4 d earlier than the control. At 30 mg L^−1^, larval occupancy further narrowed, pupal onset shifted later by ~2–3 d, and adult plateaus were reduced in breadth and height, especially for males. The 300 mg L^−1^ treatment produced the sharpest compression: larval occupancy was truncated, the pupal band was narrow, adult cohorts were small and short-lived, and no individuals were present beyond ~27–30 d. To complement the *Sₓⱼ* trajectories, stage-wise survival rates for each treatment are summarized in Table S1.

### 3.5. Life Table Parameters of T. absoluta Under MSN Exposure

The population growth parameters of *T. absoluta* were significantly affected by mesoporous silica nanoparticle (MSN) treatments in a dose-dependent manner (Table 1). The intrinsic rate of increase (r) showed a marked decline from 0.21 ± 0.01 d^−1^ in the control group to 0.07 ± 0.02 d^−1^ at 300 mg L^−1^. A corresponding decrease was observed in the finite rate of increase (λ), which dropped from 1.23 ± 0.02 d^−1^ to 1.07 ± 0.006 d^−1^ across the same gradient. Similarly, the net reproductive rate (R_0_) was highest in the control (93.7 ± 23.18 offspring/female) and decreased sharply with increasing MSN concentrations, reaching 7.1 ± 3.57 at 300 mg L^−1^. The gross reproductive rate (GRR) followed a parallel trend, decreasing from 98.93 ± 24.06 to 18.49 ± 7.80. The mean generation time (T) was prolonged under MSN stress, increasing from 21.76 ± 0.32 days in the control to a peak of 30.24 ± 0.41 days at 30 mg L^−1^. Fecundity per female showed a significant reduction, from 208.22 ± 3.43 eggs/female in the control to 47.33 ± 1.11 at 300 mg L^−1^. Additionally, the female percentage (Nf/F) declined with increasing MSN concentrations, from 0.45 ± 0.11 in the control group to 0.15 ± 0.08 at the highest dose, indicating a potential disruption in sex-specific survival or reproductive allocation.

### 3.6. Age–Stage-Specific Developmental Time

The developmental durations of *T. absoluta* exhibited significant variation in response to MSN exposure, as presented in Table 2. The egg stage duration increased slightly from 3.4 ± 0.11 days in the control group to 3.75 ± 0.14 and 3.70 ± 0.10 days at 30 mg L^−1^ and 300 mg L^−1^ MSN concentrations, respectively, indicating a mild delay in embryonic development. A marked dose-dependent extension in larval development was recorded, with the duration rising from 7.4 ± 0.11 days in the control to 11.80 ± 0.19 days at 300 mg L^−1^, suggesting progressive retardation in larval growth under higher MSN exposure. Pupal duration also increased with increasing MSN concentration, reaching a peak of 7.50 ± 0.16 days at 30 mg L^−1^; however, a slight reduction was observed at 300 mg L^−1^ (6.62 ± 0.18 days). Adult longevity was highest in the control group, at 13.63 ± 0.66 days, and decreased significantly at higher doses, reaching 9.50 ± 0.30 days at 300 mg L^−1^. The total pre-adult period exhibited a clear extension under MSN treatment, increasing from 16.37 ± 0.21 days in the control to 22.62 ± 0.31 days at the highest concentration, indicating a general delay in developmental progression across all immature stages.

### 3.7. Age–Stage-Specific Reproductive Value (Vₓⱼ) of T. absoluta

**Figure 5:**
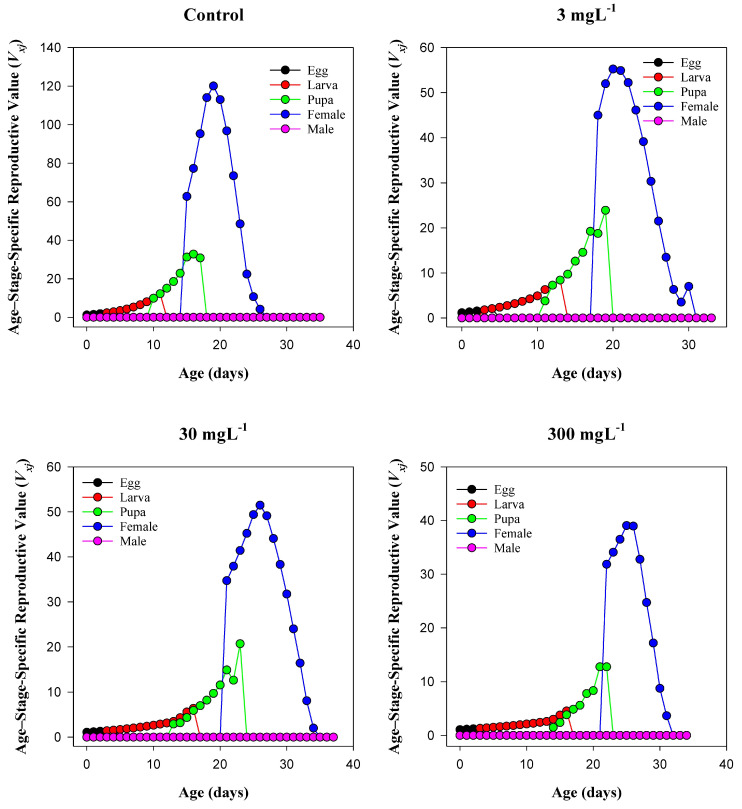
Age–stage reproductive value (*vₓⱼ*) of *T. absoluta* under mesoporous silica nanoparticle (MSN) treatments (0, 3, 30, 300 mg L^−1^). Axes: x = age (days); y = vₓⱼ. Stages shown: egg, larva, pupa, female adult, male adult.

The age–stage-specific reproductive value (*Vₓⱼ*) of *T. absoluta* exhibited substantial changes in response to increasing concentrations of mesoporous silica nanoparticles (MSNs), as illustrated in Figure 5. In the control group, the peak reproductive value reached approximately 120 around day 18, with a rapid rise beginning in the late pupal stage and extending into early adulthood. This pattern represents optimal reproductive timing and capacity under non-stressed conditions. Exposure to 3 mg L^−1^ MSNs resulted in a notable reduction in peak *Vₓⱼ* to ~50, with the apex delayed to ~days 20–21, indicating a moderate impact on reproductive development and scheduling. At 30 mg L^−1^, reproductive potential was further suppressed, with the peak *Vₓⱼ* value slightly higher (~55) than at 3 mg L^−1^, but occurring later, around day 26, and displaying a broader and more flattened trajectory, suggesting delayed reproductive maturity and decreased fecundity. The highest MSN concentration (300 mg L^−1^) resulted in the lowest reproductive value (~35), with the peak occurring near day 24 and exhibiting a narrow, compressed curve. This indicates strong inhibition of reproductive performance and condensed reproductive duration. Across all treatments, male reproductive values remained consistently negligible, reaffirming the sexually dimorphic nature of this species’ life history. Overall, MSN exposure induced a clear, dose-dependent suppression of reproductive potential in *T. absoluta*.

### 3.8. Reproductive Capacity, Pre-Oviposition Periods, and Survivorship

MSN exposure significantly affected the reproductive traits and developmental timing of *T. absoluta* in a dose-dependent manner (Table 3). The number of females (Fn) and reproductive females (RepF) declined markedly with increasing MSN concentration, dropping from 9.00 ± 2.22 in the control to 3.00 ± 1.51 at 300 mg L^−1^, indicating a strong suppression of female emergence and reproduction. The number of males varied inconsistently across treatments, with a pronounced drop at 3 mg L^−1^ (1.00 ± 2.19) and a recovery at higher doses, although still lower than the control (10.00 ± 2.23). The adult pre-oviposition period (APOP) ranged from 1.14 ± 0.14 days at 3 mg L^−1^ to 3.75 ± 0.24 days at 30 mg L^−1^, suggesting that lower doses may expedite reproductive onset, while higher concentrations delay it. The total pre-oviposition period (TPOP) was significantly prolonged in MSN-treated groups, extending from 18.44 ± 0.33 days in the control to 26.00 ± 0.40 days at 30 mg L^−1^, indicating delayed reproductive maturity. Preadult survivorship (Sa) showed a declining trend, decreasing from 0.95 ± 0.05 in the control to 0.40 ± 0.11 at 300 mg L^−1^, highlighting increased juvenile mortality under nanoparticle stress. The number of oviposition days was highest in the 3 mg L^−1^ group (9.14 ± 0.26), followed by a decline at higher concentrations, with the shortest oviposition period (5.67 ± 0.31 days) recorded at 300 mg L^−1^, indicating reproductive impairment at elevated MSN levels.

### 3.9. Age-Specific Survival and Fecundity Trends

**Figure 6:**
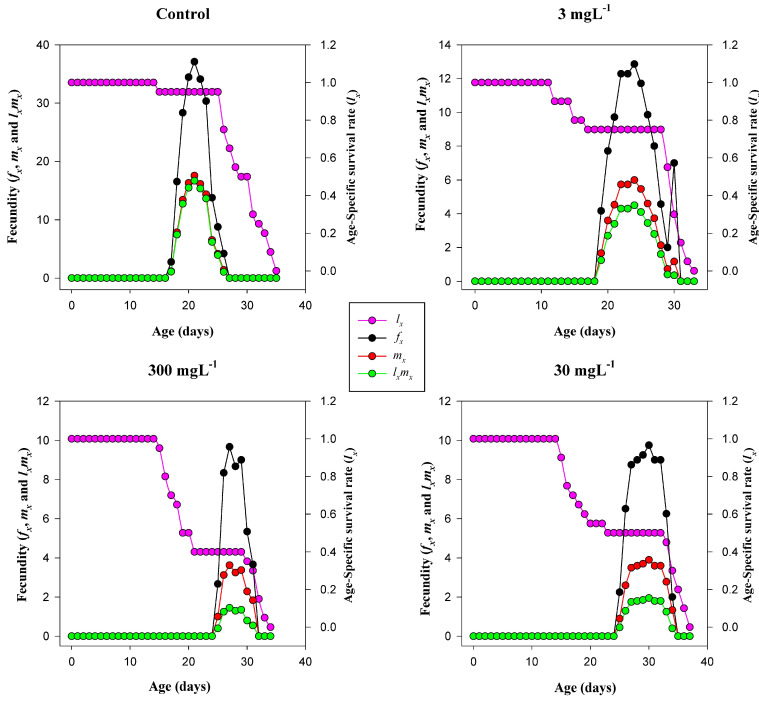
Age-specific survival (*lₓ*), age-specific fecundity (*fₓ*), age-specific maternity (*mₓ*), and net maternity (*lₓmₓ*) of *T. absoluta* under mesoporous silica nanoparticle (MSN) treatments (0, 3, 30, 300 mg L^−1^). Axes: x = age (days); y = *lₓ* (proportion) and *fₓ, mₓ, lₓmₓ* (eggs, female^−1^, day^−1^).

The reproductive and survival patterns of *T. absoluta* showed marked alterations under MSN exposure (Figure 6). In the control group, the age-specific fecundity (*f_x_*) reached its peak at approximately 35 eggs per female around day 20, coinciding with a high age-specific survival rate (*l_x_* ≈ 1.0). The net maternity function (*l_x_m_x_*) mirrored this pattern, indicating robust reproductive potential under untreated conditions. At 3 mg L^−1^, fecundity declined substantially, peaking near 12 eggs per female, with a delayed onset of reproduction around day 20. Although survival remained relatively high in the early stages, the net maternity values were significantly reduced compared to the control. In the 30 mg L^−1^ group, reproductive output further diminished, with fecundity peaking at approximately 8 eggs and compressed within a narrow reproductive window (days 26–32). The net maternity curve showed a corresponding reduction, despite moderate survivorship. At 300 mg L^−1^, both fecundity and survival were severely suppressed. Peak fecundity dropped below 6 eggs, and reproduction occurred briefly under lower survival probabilities (*l_x_* ≈ 0.4), indicating pronounced MSN-induced reproductive inhibition at the highest concentration.

### 3.10. Age–Stage Life Expectancy (e_xj_)

**Figure 7:**
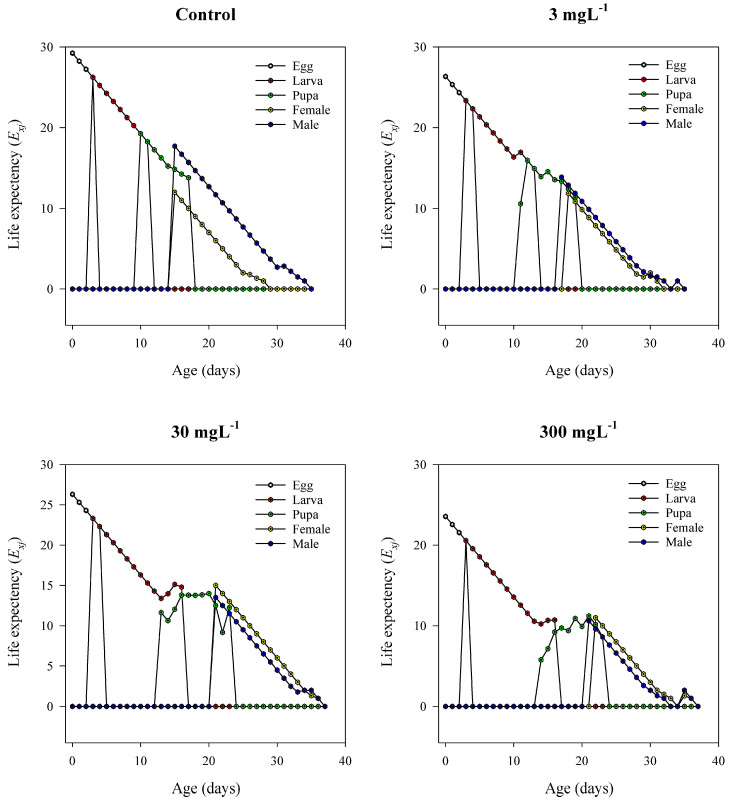
Age–stage life expectancy (*eₓⱼ*; days) of *T. absoluta* on tomato plants under mesoporous silica nanoparticle (MSN) treatments (0, 3, 30, 300 mg L^−1^). Axes: x = age (days); y = *eₓⱼ* (days).

The age–stage life expectancy curves of *T. absoluta* revealed substantial shifts in survival potential upon exposure to mesoporous silica nanoparticles (MSNs) (Figure 7). In the control group, individuals at the egg stage displayed the highest initial life expectancy (~28–30 days), which declined gradually with age. Adult females maintained higher life expectancy values than males throughout their lifespan, and the overall trajectory was smooth, reflecting stable population dynamics. At 3 mg L^−1^, the life expectancy patterns remained generally comparable to the control, but with slight irregularities in the larval and pupal phases, indicative of mild sublethal effects. Exposure to 30 mg L^−1^ MSNs resulted in a noticeable decline in *e_xj_* across all developmental stages. Early life stages showed more fluctuations, and adult females exhibited reduced longevity compared to the control. At the highest concentration (300 mg L^−1^), life expectancy decreased sharply, with significant instability observed throughout the life cycle. Egg and larval stages experienced abrupt drops in *e_xj_*, and the adult stages—particularly of females—demonstrated substantially shortened lifespans. These trends confirm that MSN treatments, especially at 30 mg L^−1^ and 300 mg L^−1^, severely compromise the survival potential and expected lifespan of *T. absoluta* in a dose-dependent manner.

### 3.11. Digestive Enzyme Activity in T. absoluta

**Figure 8:**
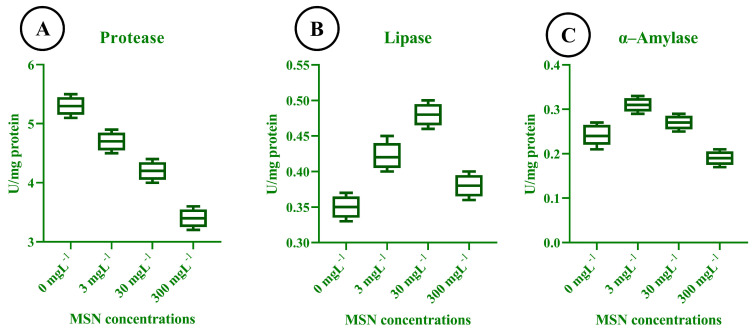
Digestive enzyme activities of *T. absoluta* larvae under mesoporous silica nanoparticle (MSN) treatments (0, 3, 30, 300 mg L^−1^). Panels: (**A**) protease, (**B**) lipase, (**C**) α-amylase; activities expressed as U mg^−1^ protein. Box–whisker plots show median, interquartile range (IQR), and range (n = 3 biological replicates per treatment).

The digestive enzyme activity of *T. absoluta* larvae exhibited marked alterations in response to different concentrations of mesoporous silica nanoparticles (MSNs), indicating dose-dependent physiological responses. Protease activity (Figure 8A) was significantly reduced with increasing MSN concentration, declining from a mean of approximately 5.5 U/mg protein in the control group to below 4.0 U/mg protein at 300 mg L^−1^. This downward trend suggests a substantial inhibition of proteolytic function under nanoparticle stress. In contrast, lipase activity (Figure 8B) initially increased slightly at 3 mg L^−1^ and peaked at 30 mg L^−1^, with an average value of approximately 0.48 U/mg protein. However, at the highest concentration of 300 mg L^−1^, lipase activity decreased, indicating that moderate MSN exposure may temporarily stimulate lipid digestion, followed by inhibition at toxic levels. α–amylase activity (Figure 8C) exhibited its highest value at 3 mg L^−1^ (~0.31 U/mg protein), followed by a progressive decline at 30 mg L^−1^ (~0.29 U/mg protein) and 300 mg L^−1^ (~0.27 U/mg protein). This pattern suggests that a low concentration of MSNs may slightly stimulate carbohydrate digestion, whereas higher concentrations result in a gradual reduction in enzymatic activity.

### 3.12. Enzymatic Response to MSN Treatments

**Figure 9:**
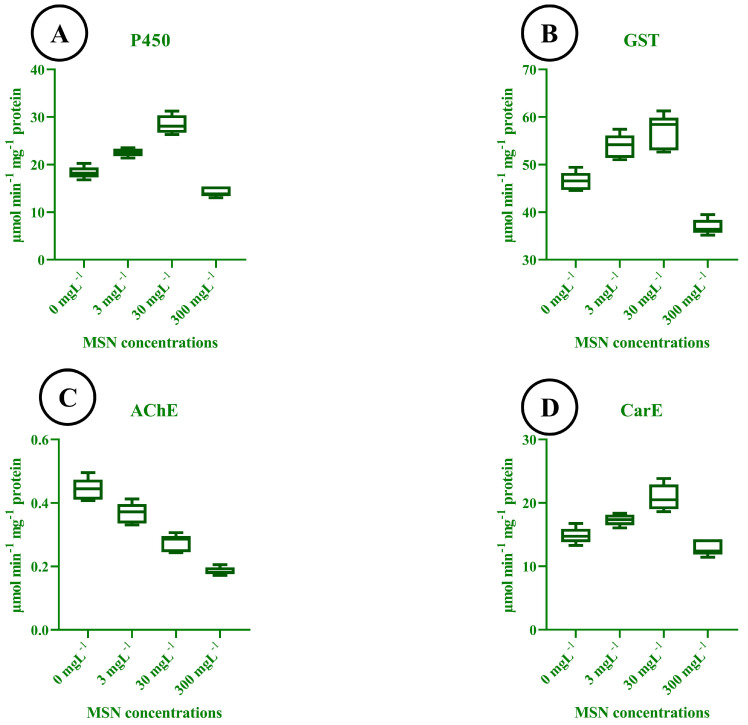
Detoxification and neural enzyme activities in *T. absoluta* larvae after exposure to MSNs (0, 3, 30, 300 mg L^−1^). (**A**) Cytochrome P450 (μmol·min^−1^·mg^−1^ protein). (**B**) Glutathione S-transferase (μmol·min^−1^·mg^−1^ protein). (**C**) Acetylcholinesterase (μmol·min^−1^·mg^−1^ protein). (**D**) Carboxylesterase (μmol·min^−1^·mg^−1^ protein). Box–whisker plots show median, interquartile range (IQR), and range (n = 3 biological replicates per treatment).

The activity of key detoxification and neural enzymes in *T. absoluta* larvae varied significantly in response to different concentrations of mesoporous silica nanoparticles (MSNs). Cytochrome P450 activity (Figure 9A) increased progressively from the control to the 30 mg L^−1^ treatment, reaching its highest value at approximately 32 μmol·min^−1^·mg^−1^ protein. However, a marked decline in P450 activity was observed at 300 mg L^−1^, indicating potential enzymatic inhibition at elevated MSN concentrations. Glutathione S-transferase (GST) activity (Figure 9B) followed a similar trend. GST levels rose from ~45 μmol·min^−1^·mg^−1^ protein in the control group to ~63 μmol·min^−1^·mg^−1^ protein at 30 mg L^−1^, before decreasing sharply at 300 mg L^−1^. This pattern reflects an initial induction of detoxification processes followed by suppression at high nanoparticle exposure. Acetylcholinesterase (AChE) activity (Figure 9C) declined consistently across all treatments. The highest activity (~0.52 μmol·min^−1^·mg^−1^ protein) was observed in the control group, while a substantial reduction was noted at 300 mg L^−1^ (~0.18 μmol·min^−1^·mg^−1^ protein), indicating an increase in neurotoxic stress with higher MSN concentrations. Carboxylesterase (CarE) activity (Figure 9D) was also affected by MSN exposure. Activity peaked at 30 mg L^−1^ (~26 μmol·min^−1^·mg^−1^ protein), showing enhanced esterase-mediated detoxification capacity, but decreased significantly at 300 mg L^−1^, likely due to systemic toxicity or metabolic disruption. These profiles indicate dose-dependent enzyme modulation: P450, GST, and CarE are induced at low to moderate concentrations (3–30 mg L^−1^) but are suppressed at 300 mg L^−1^, whereas AChE declines monotonically across doses, consistent with increasing neurotoxic stress.

### 3.13. Correlation Analysis of Plant and Insect Parameters

**Figure 10:**
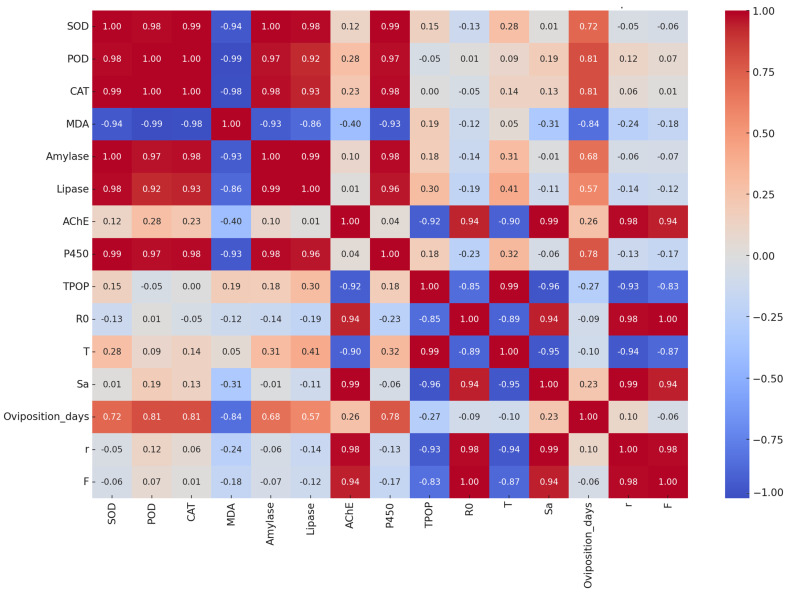
Pearson correlation matrix between plant physiological/biochemical variables and demographic parameters under MSN treatments (0, 3, 30, 300 mg L^−1^). The color scale indicates correlation coefficients from −1 to +1.

To explore the potential link between tomato physiological status and *T. absoluta* performance under MSN exposure, a Pearson correlation analysis was conducted using treatment-wise averages. The study revealed several notable associations between plant oxidative stress markers and insect enzymatic and demographic parameters (Figure 10). Superoxide dismutase (SOD), peroxidase (POD), and catalase (CAT) activities in tomato leaves showed strong positive correlations with digestive (amylase, lipase) and detoxifying (cytochrome P450) enzymes in *T. absoluta* (r ≥ 0.96). Specifically, SOD correlated strongly with P450 (r = 0.99) and lipase (r = 0.98), while CAT showed a similar pattern with amylase (r = 0.98) and lipase (r = 0.93). These trends suggest that elevated antioxidant activity in the host plant may enhance metabolic activity in the insect, possibly as a compensatory response to altered nutritional or chemical profiles of the treated foliage.

In contrast, malondialdehyde (MDA), a marker of lipid peroxidation in tomato, exhibited strong negative correlations with insect digestive enzymes—amylase (r = –0.93) and lipase (r = –0.86)—as well as reproductive traits such as oviposition duration (r = –0.84). These relationships indicate that oxidative damage in plant tissue may impair insect digestion and reproduction. POD and CAT were also positively correlated with oviposition duration (r = 0.81), suggesting that moderate MSN-induced plant responses may prolong the reproductive period of *T. absoluta*. However, antioxidant enzyme activities in the plant showed weak or negligible correlations with key insect demographic traits such as intrinsic rate of increase (r), fecundity (F), and net reproductive rate (R_0_), indicating that the effects of plant oxidative responses on overall pest fitness may be indirect or secondary. These associations are correlational and do not, by themselves, establish causal relationships between MSN exposure, physiological traits, and demographic endpoints.

## 4. Discussion

This study provides a comprehensive insight into the biochemical, physiological, and demographic responses of *T. absoluta* to mesoporous silica nanoparticles (MSNs). MSN exposure triggered oxidative damage, digestive enzyme disruption, and reproductive impairment. Together, these stress responses reduced both survival and fecundity, leading to strong population-level effects.

At lower concentrations (3 and 30 mg L^−1^), activities of superoxide dismutase (SOD), catalase (CAT), and peroxidase (POD) increased significantly, while malondialdehyde (MDA) levels decreased—hallmarks of a functional oxidative defense in response to MSN treatment. Similar enhancements in plant antioxidant enzymes have been documented following exposure to diverse nanoparticles [e.g., CAT, POD, and SOD elevation in okra under NP stress] [48] and responses to TiO_2_ and SiO_2_ NPs [increased CAT and POD with concurrent MDA reduction] [49]. Specifically, these enzymes act to alleviate lipid peroxidation and maintain cellular redox balance under abiotic stress [50]. However, at 300 mg L^−1^, antioxidant activities declined sharply while MDA increased, indicating a collapse of the oxidative defense system under high nanoparticle stress—a pattern consistent with reports of oxidative damage at excessive NP concentrations [48]. Pearson correlation analysis further confirmed strong negative associations between MDA and both CAT and POD, underscoring the inverse relationship between oxidative damage and antioxidant capacity. Moreover, elevated plant MDA was correlated with reduced insect digestive enzyme function and reproductive metrics, suggesting that oxidative stress in the host plant may impair *T. absoluta* performance—aligning with findings that plant oxidative physiology influences herbivore fitness [51]. The correlations reported here do not imply causation; targeted experiments would be necessary to demonstrate causal links.

MSN exposure also interferes with metabolic function through significant alterations in digestive enzyme activities. The suppression of protease activity at 300 mg L^−1^ suggests impaired protein hydrolysis. Lipase and α-amylase activities displayed a biphasic pattern: both increased at 30 mg L^−1^ but declined at 300 mg L^−1^. This suggests a transient stimulation of metabolism at moderate exposure, followed by suppression under high stress [33]. This pattern indicates that MSN-induced stress initially triggers a metabolic overcompensation, likely to meet increased energetic demands under oxidative conditions [52,53]. However, sustained exposure compromises digestive efficiency, potentially leading to energy deficits that manifest in extended developmental times, reduced fecundity, and shortened adult longevity [54,55]. The correlation trends between digestive enzymes and demographic parameters further support this interpretation. Although lipase and amylase showed only weak to moderate correlations with fecundity and net reproductive rate (R_0_), their contribution to energy homeostasis cannot be overlooked. Nutrient assimilation underpins the insect’s capacity for oviposition, metamorphosis, and defense responses, all of which were compromised at higher MSN concentrations [56].

The inhibitory effect of MSNs on acetylcholinesterase (AChE) activity highlights a significant neurotoxic dimension of nanoparticle exposure. AChE is essential for synaptic signal transmission, and its decline correlates strongly with reductions in fecundity and adult performance [57,58]. The consistent downregulation of AChE at increasing MSN doses suggests disruption in neuronal communication, possibly leading to behavioral anomalies or impaired physiological coordination [59]. Simultaneously, detoxification enzyme profiles (cytochrome P450, glutathione S-transferase [GST], and carboxylesterase [CarE]) exhibited a dose-dependent pattern of initial upregulation followed by enzymatic suppression at a concentration of 300 mg L^−1^. This trend implies an inducible metabolic detoxification response that becomes overwhelmed at toxic concentrations [60]. At 30 mg L^−1^, the peak activity of these enzymes may contribute to partial tolerance against oxidative and xenobiotic stress. However, at 300 mg L^−1^, the system fails, reflecting either direct enzyme inhibition or systemic physiological collapse. These mechanistic insights collectively suggest that MSNs disrupt redox balance, digestion, and neural signaling in a concentration-dependent manner, with severe consequences at higher doses [61].

The life table analysis reveals that MSN exposure has a significant impact on the population-level fitness of *T. absoluta*. Key demographic indicators—including intrinsic rate of increase (r), net reproductive rate (R_0_), and preadult survivorship (Sa)—declined sharply with increasing nanoparticle concentrations [62]. The intrinsic rate of increase decreased from 0.21 d^−1^ in the control to 0.07 d^−1^ at 300 mg L^−1^, indicating suppressed reproductive potential and reduced population growth capacity. Similarly, the net reproductive rate fell from 93.7 to just 7.1 offspring per female under the same conditions. The steep reduction in gross reproductive rate (GRR) and fecundity per female further reinforces the long-term destabilization of population dynamics due to MSN-induced physiological stress [63,64]. Developmental time is a sensitive indicator of sublethal stress. Across all life stages—egg, larva, pupa, and preadult—the exposure to MSNs induced stage-specific delays [65]. Larval duration showed the most pronounced extension, increasing by over 4 days at 300 mg L^−1^ relative to the control. This delay may stem from digestive suppression, reduced feeding efficiency, or altered hormonal regulation in response to stress. Prolonged developmental periods increase vulnerability to predation, parasitism, and environmental hazards, thereby compounding population-level impacts [66]. Age–stage-specific survival curves (*Sₓⱼ*) and reproductive values (*Vₓⱼ*) showed marked distortions under MSN exposure, particularly at 300 mg L^−1^, where the lifespan and reproductive contribution of females were significantly curtailed. These patterns align with the life expectancy data, which demonstrated substantial reductions in survival potential across all developmental stages [22]. The cumulative sublethal and lethal effects of MSN exposure compromise life history traits and reproductive continuity in *T. absoluta*, characterized by distorted survival and reproduction patterns—particularly at high concentrations (e.g., 300 mg L^−1^) [67].

The toxicological trends observed in this study are consistent with previous reports on the impacts of nanoparticles in insect systems. Zinc oxide (ZnO) and titanium dioxide (TiO_2_) nanoparticles have been shown to induce oxidative stress, developmental delays, and reproductive impairments in *S. litura*, *H. armigera*, and other lepidopterans [22,68,69,70]. Similar to our findings with MSNs, these nanoparticles disrupt redox homeostasis and metabolic function, often resulting in enzyme modulation and survival reduction [71,72]. However, MSNs appear to exert a broader spectrum of disruption, simultaneously affecting antioxidant systems, digestive enzymes, neural regulation, and demographic parameters in a unified manner. Unlike metal oxide nanoparticles, which often exert localized oxidative effects, MSNs, due to their high surface area, porosity, and silica-based reactivity, interact with multiple physiological systems [28,73]. This multi-targeted mode of action underscores the uniqueness of MSNs in pest management research. The combination of oxidative imbalance, digestive impairment, and neurotoxicity establishes MSNs as a potent but complex toxicant. These multifaceted disruptions reflect a more holistic stress profile that is particularly detrimental to pest species like *T. absoluta*, which rely on high reproductive turnover and rapid development for population maintenance [15,74,75].

The dose-dependent effects of MSNs highlight their promise as candidate nanoinsecticides. At moderate concentrations (30 mg L^−1^), MSNs significantly disrupted reproductive and developmental traits without inducing excessive mortality, suggesting a potential for population suppression with minimal ecological disruption. Their silicate-based structure, general biocompatibility, and ease of surface modification provide opportunities for formulating with targeted release mechanisms, thereby improving field-level specificity and minimizing non-target effects. Despite their potential, several limitations must be addressed before they can be applied in the field. The phytotoxicity observed at 300 mg L^−1^ suggests a narrow margin of safety between effective and harmful doses. Additionally, the environmental persistence, bioaccumulation potential, and long-term effects on non-target organisms, including pollinators and natural enemies, remain underexplored. The development of controlled-release formulations, biodegradable carriers, or surface-functionalized MSNs may help mitigate these concerns. The broader ecological impacts of using nanomaterials in agriculture—such as disruption of soil microbiota, trophic transfer, or unintended ecological cascades—necessitate rigorous risk assessments. As nanotechnology continues to evolve within the pest management sector, an integrated understanding of nanoparticle–insect–plant interactions will be critical to ensuring both efficacy and environmental sustainability.

## 5. Conclusions

This study demonstrates that mesoporous silica nanoparticles (MSNs) induce significant physiological and biochemical stress on *T. absoluta* in a dose-dependent manner, with important implications for both pest control and plant health. Among the tested concentrations, 30 mg L^−1^ was identified as the optimal dose—effectively disrupting pest development and reproduction while maintaining an acceptable physiological status in the host tomato plant. At this concentration, *T. absoluta* exhibited disrupted digestive enzyme balance, impaired nutrient assimilation, and prolonged larval and preadult developmental periods. The demographic data further revealed a marked reduction in the intrinsic rate of increase (r), net reproductive rate (R_0_), fecundity, and reproductive days, indicating suppressed population growth. In parallel, antioxidant enzyme activity in the tomato plant (SOD, CAT, POD) was significantly enhanced at 30 mg L^−1^, accompanied by a reduction in malondialdehyde (MDA) levels, suggesting a functional oxidative defense response in the plant. These changes in plant physiology were correlated with key insect traits, highlighting the role of host-mediated effects in shaping pest responses. In contrast, the highest concentration (300 mg L^−1^) compromised both plant and insect physiology, suppressing antioxidant enzyme activity in the plant, increasing lipid peroxidation, and leading to severe reductions in pest survival, enzymatic function, and reproductive output. However, the phytotoxic effects observed at this dose limit its practical utility in agricultural settings. By influencing multiple biological pathways—plant oxidative signaling, insect digestion, neurophysiology, and detoxification—MSNs at 30 mg L^−1^ provide a balanced and targeted approach to pest suppression without compromising crop viability. These findings support the integration of MSNs into nano-enabled pest management strategies, particularly when applied at carefully optimized concentrations. Future research should focus on developing controlled-release formulations, evaluating environmental safety, and validating efficacy under field conditions to facilitate the safe deployment of MSN-based biopesticides in sustainable agriculture. These findings suggest that mesoporous silica nanoparticles can be integrated into integrated pest management (IPM) strategies as a safer alternative to conventional insecticides. Validation under greenhouse and field conditions, together with non-target assessments, will be essential to confirm their practical applicability and long-term safety.

## Figures and Tables

**Table 1:** Table of bootstrapping results of population parameters of *T. absoluta* fed on tomato leaves treated with mesoporous silica nanoparticles.

	0 mg L^−1^	3 mg L^−1^	30 mg L^−1^	300 mg L^−1^
Intrinsic rate of increase (r)	0.21 ± 0.01	0.14 ± 0.01	0.09 ± 0.02	0.07 ± 0.02
Finite rate of increase (λ)	1.23 ± 0.02	1.16 ± 0.02	1.09 ± 0.02	1.07 ± 0.006
Net reproduction rate (R_0_)	93.7 ± 23.18	33.15 ± 8.89	14.35 ± 6.28	7.1 ± 3.57
Mean generation time (T)	21.76 ± 0.32	24.23 ± 0.32	30.24 ± 0.41	28.83 ± 0.41
Gross reproduction rate (GRR)	98.93 ± 24.06	45.09 ± 12.95	29.51 ± 11.03	18.49 ± 7.80
Fecundity per female (F)	0.208.22 ± 3.43	97.71 ± 4.32	71.75 ± 1.22	47.33 ± 1.11
Female percentage (Nf/F)	0.45 ± 0.11	0.35 ± 0.11	0.2 ± 0.09	0.15 ± 0.08

**Table 2:** Age–stage-specific developmental time (days) of *T. absoluta* fed on tomato leaves treated with mesoporous silica nanoparticles.

	0 mg L^−1^	3 mg L^−1^	30 mg L^−1^	300 mg L^−1^
Egg (n)	3.4 ± 0.11	3.60 ± 0.15	3.75 ± 0.14	3.70 ± 0.10
Larva (days)	7.4 ± 0.11	8.45 ± 0.11	10.90 ± 0.19	11.80 ± 0.19
Pupa (days)	5.58 ± 0.11	6.40 ± 0.13	7.50 ± 0.16	6.62 ± 0.18
Adult (days)	13.63 ± 0.66	11.87 ± 0.23	13.10 ± 0.22	9.50 ± 0.30
Pre-adult (days)	16.37 ± 0.21	18.53 ± 0.21	22.00 ± 0.35	22.62 ± 0.31

**Table 3:** Table of number of females, APOP, TPOP, preadult survivorship, and oviposition days estimated by using the bootstrap method.

	0 mg L^−1^	3 mg L^−1^	30 mg L^−1^	300 mg L^−1^
Fn	9.00 ± 2.22	7.00 ± 2.13	4.00 ± 1.75	3.00 ± 1.51
RepF	9.00 ± 2.22	7.00 ± 2.13	4.00 ± 1.75	3.00 ± 1.51
Male	10.00 ± 2.23	1.00 ± 2.19	6.00 ± 2.03	5.00 ± 1.90
APOP	2.11 ± 0.11	1.14 ± 0.14	3.75 ± 0.24	3.00 ± 0.00
TPOP	18.44 ± 0.33	19.71 ± 0.28	26.00 ± 0.40	25.67 ± 0.31
Sa	0.95 ± 0.05	0.75 ± 0.10	0.50 ± 0.11	0.40 ± 0.11
Ovi-days	6.56 ± 0.17	9.14 ± 0.26	8.00 ± 0.00	5.67 ± 0.31

Fn = No. of females; RepF = No. of reproductive females; APOP = Adult pre-oviposition period; TPOP = Total pre-oviposition period; Sa = Preadult survival rate; Ovi-days = Oviposition days.

## Data Availability

Data and analysis scripts arepublicly available at Zenodo (https://doi.org/10.5281/zenodo.16925986).

## Supplementary Materials

The following supporting information can be downloaded at: https://www.mdpi.com/article/10.3390/insects16090877/s1, Supplementary Table S1: Stage-wise survival of *Tuta absoluta* under MSN treatments (%, 95% Wilson CI).

## Abbreviations

The following abbreviations are used in this manuscript:

MSN(s)	Mesoporous Silica Nanoparticle(s)
*T. absoluta*	*Tuta absoluta*
ROS	Reactive Oxygen Species
SOD	Superoxide Dismutase
CAT	Catalase
POD	Peroxidase
MDA	Malondialdehyde
P450	Cytochrome P450 Monooxygenases
AChE	Acetylcholinesterase
CarE	Carboxylesterase
GST	Glutathione S-Transferase
RWC	Relative Water Content
Pn	Photosynthetic Rate
gs	Stomatal Conductance
r	Intrinsic Rate of Increase
λ	Finite Rate of Increase
R_0_	Net Reproductive Rate
GRR	Gross Reproductive Rate
T	Mean Generation Time
APOP	Adult Pre-Oviposition Period
TPOP	Total Pre-Oviposition Period
Sa	Preadult Survivorship
fx	Age-Specific Fecundity
lx	Age-Specific Survival Rate
Vₓⱼ	Age–Stage Reproductive Value
exj	Age–Stage Life Expectancy
IPM	Integrated Pest Management
DLS	Dynamic Light Scattering
TEM	Transmission Electron Microscopy
SEM	Scanning Electron Microscopy
BET	Brunauer–Emmett–Teller (Surface Area Analysis)
XRD	X-ray Diffraction
SPAD	Soil Plant Analysis Development (Chlorophyll Meter Reading)
ANOVA	Analysis of Variance
