# Supplementary material for: Mesoporous Silica Nanoparticles Impair Physiology and Reproductive Fitness of Tuta absoluta Through Plant-Mediated Oxidative Stress and Enzymatic Disruption
Source: Insects. 2025 Aug 23;16(9):877. doi: 10.3390/insects16090877 (PMC12471041; doi:10.3390/insects16090877)
Supplement: Supplementary file 1 [file insects-16-00877-s001.zip › insects-3779831-supplementary.pdf]

Table S1. Stage-wise survival of *Tuta absoluta* under MSN treatments (%, 95% Wilson CI). *(Egg→Larva and Egg→Adult use N = 60 eggs per treatment; Larva→Pupa uses N = 60 larvae per treatment; Pupa→Adult uses N equal to the number of pupae for that treatment: 0 mg L^{-1} = 60, 3 mg L^{-1} = 45, 30 mg L^{-1} = 30, 300 mg L^{-1} = 24.)*

Treatment	Egg→Larva	Larva→Pupa	Pupa→Adult	Egg→Adult
0 mg L^{-1}	100.0 (94.0–100.0)	100.0 (94.0–100.0)	95.0 (86.3–98.3)	95.0 (86.3–98.3)
3 mg L^{-1}	100.0 (94.0–100.0)	75.0 (62.8–84.2)	100.0 (92.1–100.0)	75.0 (62.8–84.2)
30 mg L^{-1}	100.0 (94.0–100.0)	50.0 (37.7–62.3)	100.0 (88.6–100.0)	50.0 (37.7–62.3)
300 mg L^{-1}	100.0 (94.0–100.0)	40.0 (28.6–52.6)	100.0 (86.2–100.0)	40.0 (28.6–52.6)

Notes.

1. CIs are Wilson score intervals with z = 1.96.
2. Denominators reflect totals across **three biological replicates** (20 individuals each). For Pupa→Adult, the denominator is the number of pupae observed at that dose (given in the table heading).
3. Values are percentages; ranges in parentheses are the 95% CIs (same units).
